# A novel blood based triage test for colorectal cancer in primary care: a pilot study

**DOI:** 10.3399/BJGPO.2022.0077

**Published:** 2022-12-14

**Authors:** Cerys Jenkins, Freya Woods, Susan Chandler, Kym Carter, Rhys Jenkins, Andrew Cunningham, Kayleigh Nelson, Rachel Still, Jenna A Walters, Non Gwynn, Wilson Chea, Rachel Harford, Claire O'Neill, Julie Hepburn, Ian Hill, Heather Wilkes, Greg Fegan, Peter Dunstan, Dean A Harris

**Affiliations:** 1 Physics Department, College of Science, Centre for NanoHealth, Swansea University, Swansea, UK; 2 Swansea University Medical School, Swansea University, Swansea, UK; 3 Swansea Trials Unit, Swansea University, Swansea, UK; 4 Department of Laboratory Medicine, of Medical Biochemistry, Swansea Bay University Health Board, Swansea, UK; 5 Department of Colorectal Surgery, Swansea Bay University Health Board, Swansea Bay University Health Board, Morriston Hospital, Swansea, UK; 6 Research and Development Department Swansea Bay University Health Board, Institute of Life Science 2, Swansea University, Swansea, UK; 7 Public Involvement Community, Health and Care Research Wales, Cardiff, UK; 8 Briton Ferry Primary Care Centre, Neath, UK

**Keywords:** colorectal neoplasms, two-week wait, pilot projects, qualitative research, Raman spectroscopy, primary health care, general practice

## Abstract

**Background:**

The majority of colorectal cancers (CRCs) are detected after symptomatic presentation to primary care. Given the shared symptoms of CRC and benign disorders, it is challenging to manage the risk of missed diagnosis. Colonoscopy resources cannot keep pace with increasing demand. There is a pressing need for access to simple triage tools in primary care to help prioritise patients for referral.

**Aim:**

To evaluate the performance of a novel spectroscopy-based CRC blood test in primary care.

**Design & setting:**

Mixed-methods pilot study of test performance and GP focus group discussions in South Wales.

**Method:**

Patients on the urgent suspected cancer (USC) pathway were recruited for the Raman spectroscopy (RS) test coupled to machine learning classification (‘Raman-CRC’) to identify CRC within the referred population. Qualitative focus group work evaluated the acceptability of the test in primary care by thematic analysis of focus group theorising.

**Results:**

A total of 532 patients aged ≥50 years referred on the USC pathway were recruited from 27 GP practices. Twenty-nine patients (5.0%) were diagnosed with CRC. Raman-CRC identified CRC with sensitivity 95.7%, specificity 69.3% with area under curve (AUC) of 0.80 compared with colonoscopy as the reference test (248 patients). Stage I and II cancers were detected with 78.6% sensitivity. Focus group themes underlined the convenience of a blood test for the patient and the test’s value as a risk assessment tool in primary care.

**Conclusion:**

The findings support this novel, non-invasive, blood-based method to prioritise those patients most likely to have CRC. Raman-CRC may accelerate access to diagnosis with potential to improve cancer outcomes.

## How this fits in

Current CRC referral pathways are resource intensive with a low conversion rate. There is currently a lack of effective triage tests for suspected CRC in primary care. The Raman-CRC blood test is highly sensitive for all-stage (95.7%) and stage I–II (78.6%) CRC detection. GP focus groups agreed that the test would help increase early stage cancer detection in primary care.

## Introduction

CRC is the second largest cause of cancer-related deaths worldwide.^1^ The majority (54%) of CRC in the UK is diagnosed through primary care consultation and referral.^2^ Patients satisfying strict clinical referral criteria can be referred from primary care on the USC or two-week wait (2WW) pathway for further investigations and treatment within a 62-day target.^3^

The strict referral criteria fail to take into account the GP’s ‘gut instinct’ for serious pathology and have a low cancer conversion rate as the USC symptom profile is based on a minimum positive predictive value (PPV) for cancer of just 3%. Despite demand for colonoscopy doubling over the past 5 years,^4^ the current USC pathway has failed to detect CRC earlier nor changes the outcomes of CRC,^5^ with the UK having one of the poorest CRC survival records in Europe.

Increasing numbers of patients with lower gastrointestinal (GI) symptoms are presenting to primary care. With the limitations of endoscopy resource, there is a need for simple triage tests to be available to GPs to help risk-manage presenting patients, particularly given the rapid increase in early age of onset CRC.

Faecal immunochemical testing (FIT) for faecal haemoglobin (*f*-Hb) was introduced in 2017 for low-risk symptom triage in primary care (National Institute for Health and Care Excellence [NICE] Diagnostics guidance [DG30])^6^ with evidence growing for its use in high-risk symptoms meeting NICE NG12 criteria. The recent NICE FIT study reported test sensitivity of 90.9% and specificity of 83.5% at cut-off of 10 µg/g *f*-Hb.^7^ FIT may not be the ideal triage tool for primary care use given its low return rate^8,9^ (just 62% of patients in the NICE FIT study), its lack of cancer specificity, and its lack of approval for rectal bleeding,^10^ which is the commonest presenting symptom.

There is much recent interest in the use of artificial intelligence (AI) to identify cancer more efficiently in primary care, such as the ‘C the Signs’ and ‘Pinpoint-AI’ applications. The authors have developed a simple blood test that uses RS to measure cancer-related molecular species (proteins, nucleic acids, lipids) in serum to produce a cancer-specific ‘biochemical fingerprint’. An AI-algorithm analyses the spectral output and classifies the patient into either high or low likelihood of CRC. This test could help GPs to identify and prioritise patients with suspected cancer for further investigation as a referral decision support tool.^11^

This article presents the results of a pilot application of the Raman-CRC model to detect CRC in a cohort of patients meeting USC referral criteria from a primary care setting. This study presents a mixed-methods approach, considering the utility of a Raman-CRC blood test to streamline the referral pathway for patients with suspected cancer, and explores its potential to translate into a clinical setting.

## Method

### Study design 

A prospective cohort pilot study was undertaken to evaluate the performance of Raman-CRC in primary care to triage need for referral and diagnostic testing for CRC, as outlined by the Detecting Cancer Early Setting Partnership.^12^ This work was performed as a phase-2 evaluation of clinical test performance (analytic validity in intended setting) in accordance with the CanTest framework.^13^ Results of Raman-CRC were compared with final patient diagnosis after USC investigations to determine sensitivity and specificity in an enriched symptomatic primary care population.

The study was conducted within Swansea Bay University Health Board (SBUHB) primary care practices and managed by Swansea Trials Unit. Patient demographics, current USC pathway timelines, and final diagnosis were obtained from electronic patient records and recorded in a REDCap database.^14^ Interval cancers were captured up to 9 months after diagnosis. Results were reported according to QUADAS-2 standards.

**Figure 1.**
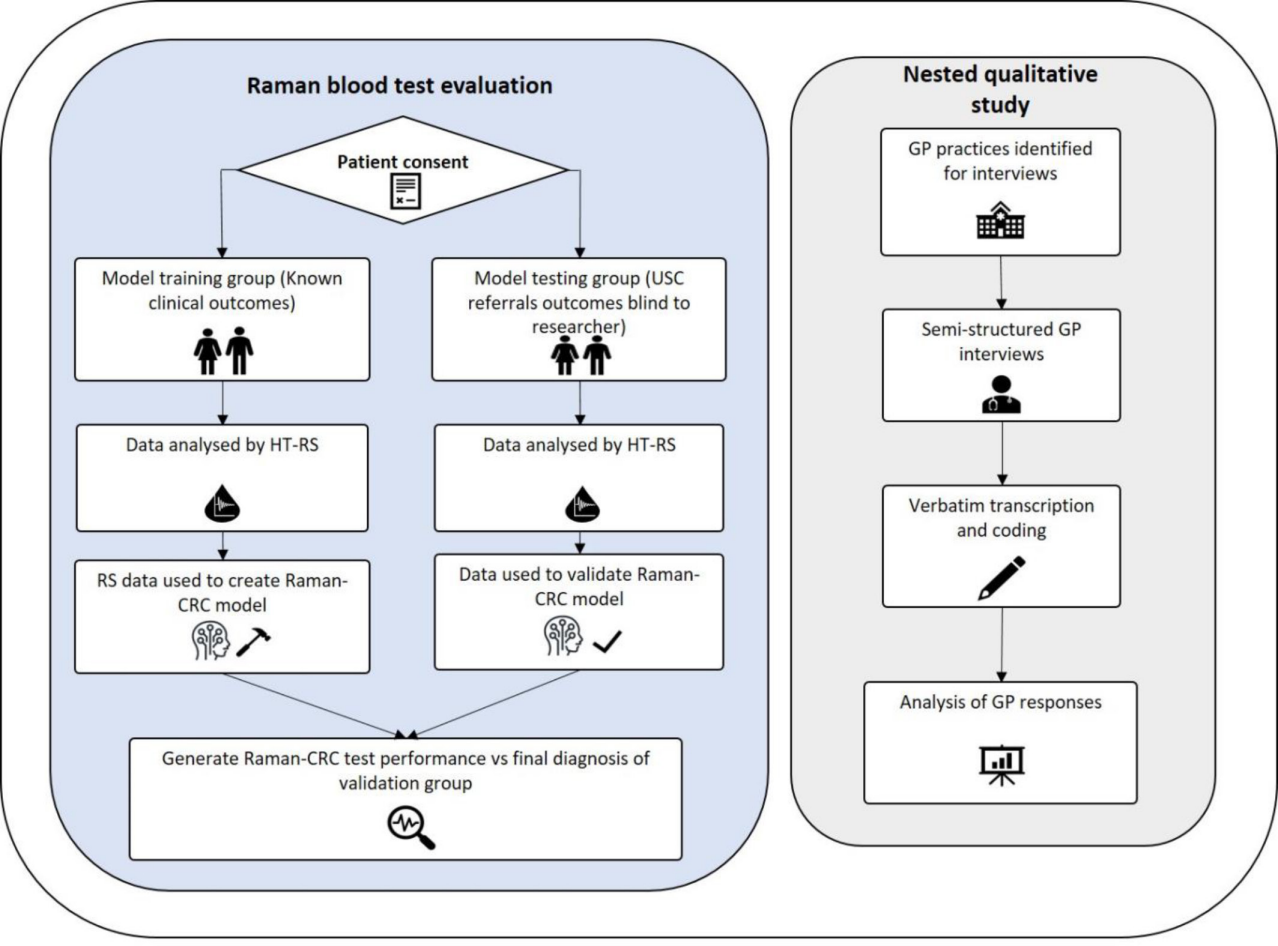
Study design. The mixed -methods prospective clinical validation study incorporated a retrospective cohort analysis to build the Raman-model, the prospective study for clinical validation, and a nested qualitative study for investigating attitudes of the test in primary care. USC = urgent suspected cancer; CRC = colorectal cancer. USC = urgent suspected cancer.

A nested qualitative study was performed and reported according to the COnsolidated criteria for REporting Qualitative research (COREQ) checklist. This involved semi-structured focus group discussions with GP practices^15^ to explore attitudes towards the current USC pathway and the potential uses of Raman-CRC in primary care (Figure 1).

### Participants

Eligible participants were aged ≥50 years and had presented to their GPs with high-risk symptoms raising suspicion of CRC as per NICE guidelines (NG12).^3^ 

### Blood sample preparation  

Fasted venous blood samples were centrifuged, aliquoted, and stored at -80°C before batch analysis.

### Statistical analysis

#### Sample size planning

The study was designed to estimate test performance of the Raman-CRC model in a population with a cancer prevalence representative of its intended application. A sample size of at least 75–100 patients is required as an independent blinded test set.^16^ A model sample set of 300 patients is utilised to surpass this minimum sample requirement. To provide a definitive sample size for precise determination of the performance of the analysis model, it was assumed that a 5% prevalence would require 691 recruited participants based on a specificity of 90% with absolute precision of 0.1.^17,18^

### Raman spectroscopy 

Serum samples were analysed using previously reported high-throughput (HT) Raman methodology with modifications.^11^ Serum samples were thawed before analysis; liquid serum samples (200 µL) were placed into the HT platform and analysed with a 785 nm laser using a Raman microscope.

### Raman-CRC machine learning model  

All Raman spectra underwent data pre-processing including wavenumber calibration, data binning, smoothing, background subtraction, and normalisation.^19,20^ A random forest-based machine learning model showed optimal performance and a diagnostic model was developed using a retrospective cohort of 300 patients with known clinical outcomes of CRC (histologically confirmed) or non-cancer control (normal colonoscopy) in a 50:50 split.^21^ The Raman-CRC model was internally cross-validated using a repeated 5-fold cross validation of training data to produce a preliminary AUC and sensitivity and specificity values within R.

### Prospective clinical validation study

Thirty-five GP practices within SBUHB were invited to take part in the study from 2017–2019 of which 27 practices participated (77.0%) with additional recruitment from secondary care after referral. Nine patients declined study participation, leaving 595 patients who provided blood samples at time of consent (98.5% compliance) (Supplementary Figure S1).

Analytic researchers were blinded to final diagnosis. The average cancer probability for all spectra from each patient was then aggregated to produce an overall predicted cancer probability. Any patient with a probability of ≥0.5 was classified as ‘CRC’, and <0.5 ‘non-cancer’.

### Reference standard  

The resultant decision for each patient produced by Raman-CRC was compared with final diagnosis as confirmed following colonoscopy or computed tomography (CT) colonography with histological verification. Patients who did not undergo reference standard tests or had data missing from diagnostic results were excluded. Colonoscopy was the primary reference standard. The results were analysed per investigation and reported separately as CT colonography has reduced ability to detect small polyps and flat cancers.^22,23^ Patients who were investigated with flexible sigmoidoscopy were excluded.

### Primary care interviews

Semi-structured focus groups were carried out at primary care practices in South Wales. Scenarios were presented during the focus groups to explore attitudes towards test application for different clinical situations with data on RS performance based on a previous pilot results (sensitivity 85.7%, specificity 68%; Supplementary Table S1, Supplementary Boxes S1–2). The focus groups were conducted face to face at GP practice sites (one via video-conferencing) by DAH, and were audio-recorded and transcribed verbatim before analysis.

### Qualitative analysis 

Following checked transcription, NVivo software (version 12) was used to code and analyse the transcripts. Three researchers (one male, two female) independently coded the interviews to identify potential themes and the independent analyses were merged into a final coding scheme (Supplementary Table S2).^24^ Sub-themes were generated based on consensus. 

## Results

### Development of the Raman-CRC diagnostic model

Three hundred patients were age and sex-matched using propensity score matching to develop the Raman-CRC blood test model (Supplementary Table S3). The Raman CRC model showed an area under the curve (AUC) value of 0.842 for a typical fold where an AUC between 0.8 and 0.9 is considered excellent.^25^ The model achieved a sensitivity of 70.5% and specificity of 76.8% when trained on 150 known cancers (52.0% [79/150] stage I and II, and 48.0% [72/150] stage III and IV) and 150 controls (Supplementary Figure S2).

### Prospective validation study

The study captured a wide variance of diagnoses within the 532 eligible primary care patients including non-cancer conditions, pre-cancerous polyps, and other cancer types (Supplementary Table S4). Twenty-nine patients (5.0%) were diagnosed with CRC. Patient ages were comparable between groups with a male predominance in the CRC group (Supplementary Table S5). Minimal differences in symptom frequency or routine blood results (haemoglobin, ferritin) between cancers and non-cancer diagnoses was observed, highlighting the lack of diagnostic specificity of currently used clinical features.

**Figure 2.**
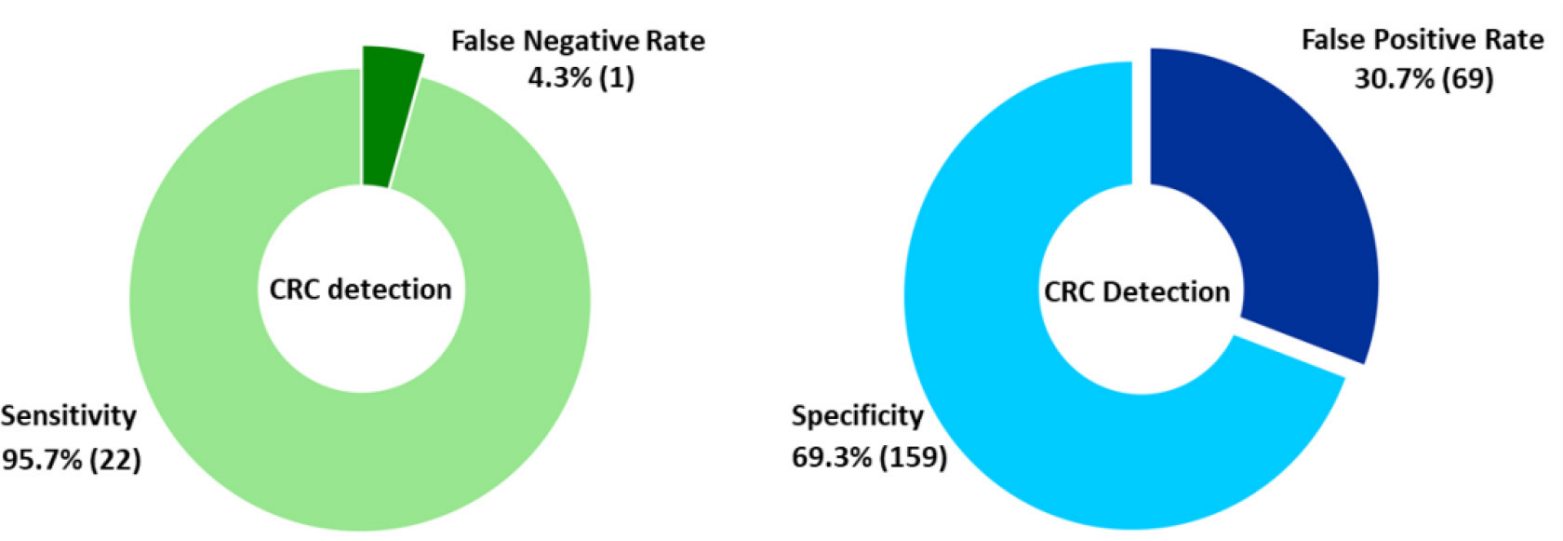
Disease prediction for the prospective validation cohort from secondary care urgent suspected cancer (USC) referral patients. Overall sensitivity, specificity, false negative rate, and false positive rate for the Raman-CRC model on a colonoscopy per-patient basis following blind analysis. CRC = colorectal cancer.

After patient exclusions, 405 patients remained with CRC or non-CRC diagnosis based on colonoscopy or CT colonography. Compared with gold standard colonoscopy, the model showed a sensitivity of 95.7% and a specificity of 69.3% (Figure 2 and Table 1).

**Table 1.** Disease prediction for the prospective validation cohort from secondary care USC referral patients. Test sensitivity and specificity (95% confidence intervals according to initial diagnostic test)

	*n*, total	Sensitivity	Specificity
Colonoscopy	248	95.7 (78.1% to 99.9%)	69.3 (63.8% to 76.1%)
Colonoscopy and CTC	405	89.7 (72.7% to 97.8%)	65.7 (60.7% to 70.5%)

CTC = computed tomography colonoscopy. USC = urgent suspected cancer.

Test performance by colonoscopy and CT colonogram combined found sensitivity of 89.7% and specificity 65.7%. The Raman blood test detected early stage CRC (UICC stage I–II) with 78.6% sensitivity and III–IV with 100% sensitivity (Table 2), comparing favourably with FIT data (Table 3).

**Table 2.** Raman-CRC model performance for different cancer stages versus FIT

	Sensitivity for UICC guidance stages			
Stage	I	II	III	IV
Raman-CRC	50% (*n* = 4)	90% (*n* = 10)	100% (*n* = 12)	100% (*n* = 3)
FIT^^27^^	73% (65%–79%)	80% (74%–84%)	82% (77%–87%)	79% (70%–86%)

CRC = colorectal cancer. FIT = faecal immunochemical test.

**Table 3.** Comparison of Raman-CRC test performance with NG12 pathway and FIT

	NICE NG12 pathway^3^	FIT (threshold 10 µg/g)^7^	Raman-CRC
Sensitivity	93	90.9	95.7
Specificity	35	83.5	69.3
AUC	n/a	0.93	0.8

AUC = area under curve. CRC = colorectal cancer. FIT = faecal immunochemical test. NICE = National Institute for Health and Care Excellence.

### Acceptability of a Raman blood test in primary care

A nested qualitative study was conducted through focus group discussion across six primary care practices and included 24 GPs. The mean meeting duration was 45 minutes (range 35–55 minutes) and followed a semi-structured question format. The following four key themes were identified from the discussions: perceptions of the current referral pathway; utility of Raman-CRC as a triage tool; utility of Raman-CRC as a diagnostic tool; and GP acceptability of Raman-CRC (summarised with quotes in Supplementary Table S7).

#### Perceptions of the current referral pathway

GPs agreed that they carefully considered appropriateness of USC referrals and were conscious of current capacity problems within secondary care. GPs also felt under pressure to get patients seen within USC pathway timeframes. They highlighted patients often experienced long waits for ‘urgent’ referrals and as such would try to *'shoehorn*' (GP 2, practice 5) patients into the USC pathway to fulfil their duty of care in a timely manner. While most GPs thought the referral criteria were very rigid making it difficult to refer patients outside of the criteria, they 'shoehorned' patients when they had clinical concerns.

#### Utility of Raman-CRC as a triage tool

GPs welcomed the proposed Raman-CRC test to help triage patients being referred and make more appropriate referral decisions. They highlighted that the test might reduce the number of unnecessary referrals and that it may be preferable for some patients, particularly when compared with faecal-based tests. It was thought the test would go some way to help GPs remove barriers to earlier diagnoses by using the test results as evidence to refer patients (as a ‘rule-in’ test rather than a ‘rule-out’ test)

GPs also highlighted other potential uses for the test and all agreed that it would be most useful in helping to provide an evidence base for, and enabling better management of, patients who had symptoms that did not meet the USC referral criteria. The test would be an acceptable method to reassure patients and reduce their anxiety:

*‘It’s very good at saying you haven't got cancer so you can be reassured.’* (GP 2, practice 1)

#### Utility of Raman-CRC as a diagnostic tool

GPs highlighted that the test has potential as a diagnostic tool in populations where invasive testing is not appropriate; for example, for frail patients it would potentially provide a diagnosis without invasive diagnostic procedures causing harm or distress:

*'it would be helpful in the ones you have a real strong feeling about but they don’t fit the criteria'* (GP1, practice 2)

GPs on the whole felt comfortable using it as a screening tool and stated they would be comfortable providing the results to patients.

#### GP acceptability of Raman-CRC

To have the confidence to use Raman-CRC routinely in primary care all agreed it needed to be adopted into local or national guidelines. However, GPs agreed that if the test were available and within the guidance then it would be well utilised:

*'If a Raman blood test was available then I would do it, and I think you would find every GP would.*' (GP 1, practice 3)

## Discussion

### Summary

The article reports the first prospective proof of concept study to analyse blood serum with label-free RS combined with machine learning as a disruptive new technology for transforming the current suspected cancer pathway for CRC. It shows early evidence that Raman-CRC has sufficient test performance for future utility in primary care as a ‘rule-in’ triage test. This could be of great value in detecting CRC in younger symptomatic patients in primary care, in whom cancer incidence is rising, and to streamline the referral pathway for diagnostic investigations.

Analysis of focus group transcripts revealed overwhelming support for the blood test and highlighted the need for a primary care-based companion test to triage primary care referrals. GP attitudes were positive towards adoption and clinical utility for a blood-based test for CRC in primary care. The projected reduction in patient anxiety was positively received. Test performance was considered acceptable even at this proof of concept stage and would be used to influence referral behaviour if routinely available.

### Strengths and limitations

This prospective test evaluation was conducted to strict guidelines with blinding of analysts to final diagnosis. Reference standard was restricted to colonoscopy as gold standard. It is recognised that the cancer event rate was small (yet representative of the USC population), which could have made the sensitivity of the Raman-CRC test appear higher. Raman-CRC specificity is currently inferior to that of FIT (69.3% versus 83.5%) but still exceeds the NICE NG12 criteria specificity of just 35%^26^ as a significant advance over the symptom-based referral route alone (Table 3). FIT was not available locally at the time of the study as a comparison group, but is to be included in follow-on studies.

### Comparison with existing literature

Raman-CRC showed superior overall sensitivity for CRC (95.7% versus 90.9%) compared with recently published FIT performance at a cut-off of ≥10 µg/g of *f*-Hb (Table 3).^7,26^ The Raman-CRC test has a sensitivity for early stage I and II CRC of 78.6%. Sensitivity by stage for FIT is poorly reported in general, although Niedermaier *et al* reported pooled sensitivity of 79–82% (95% CI = 70% to 87%) for stage III and IV cancers and 73%–80% (95% CI = 65% to 84%) for stage I and II cancers with just 40% sensitivity for T1 cancers (Table 2).^27^ Data from multi-cancer circulating deoxyribonucleic acid (DNA) blood tests suggest even worse performance for early stage CRC (67% sensitivity stage I and II),^28^ which may not be the solution to achieve the NHS Long Term Plan of detecting 75% of cancers at stage I and II by 2028.^29^

### Implications for research and practice

The International Cancer Benchmarking Partnership (ICBP) has reported the UK has the lowest survival rates for CRC, in part through differences in diagnostic pathways and referral timelines.^30,31^ More accurate and acceptable tests, such as Raman-CRC, could improve this situation. The test could be applied at first primary care consultation to avoid missed opportunities for earlier detection, particularly for early onset CRC. It may also encourage earlier help-seeking behaviour of hard-to-reach patient groups given its familarity as ‘just a blood test’.

It is recognised that this proof-of-concept model will require collection of larger datasets to train more advanced models for validation. An improved AI algorithm trained on greater numbers of cancers with inclusion of high-risk adenomas and patient metadata (demographics, symptoms, routinely available blood test results) is in development with potential for superior test performance and enhanced specificity rates. A larger cohort study evaluating Raman-CRC in combination with FIT (CRaFT study, IRAS 254366) will further develop the current AI model and measure individual and combined test accuracy with FIT. The NICE FIT study highlighted the need for a further test alongside FIT to reduce the false positives and false negatives.^7^ CRaFT will also capture symptomatic patients’ experiences and attitudes with Raman and FIT. Future work is planned to conduct a health economic cost-effectiveness analysis, impact analysis in terms of earlier detection and use of downstream resources, and qualitative analysis of patient and clinician test acceptability.

Other emerging technologies include circulating tumour (ct)DNA in plasma. Although showing promising sensitivity and specificity in advanced cancers, these technologies are not yet validated in target clinical populations with low cancer prevalence and may not be cost-effective for NHS use.^32^ Raman-CRC has discernible advantages through being a rapid, reproducible, high-throughput technology whose cost per test is minimised by its reagent-free approach. It also shows early promise in multi-cancer detection in a community rapid diagnostic clinic setting.^33^

**Figure 3.**
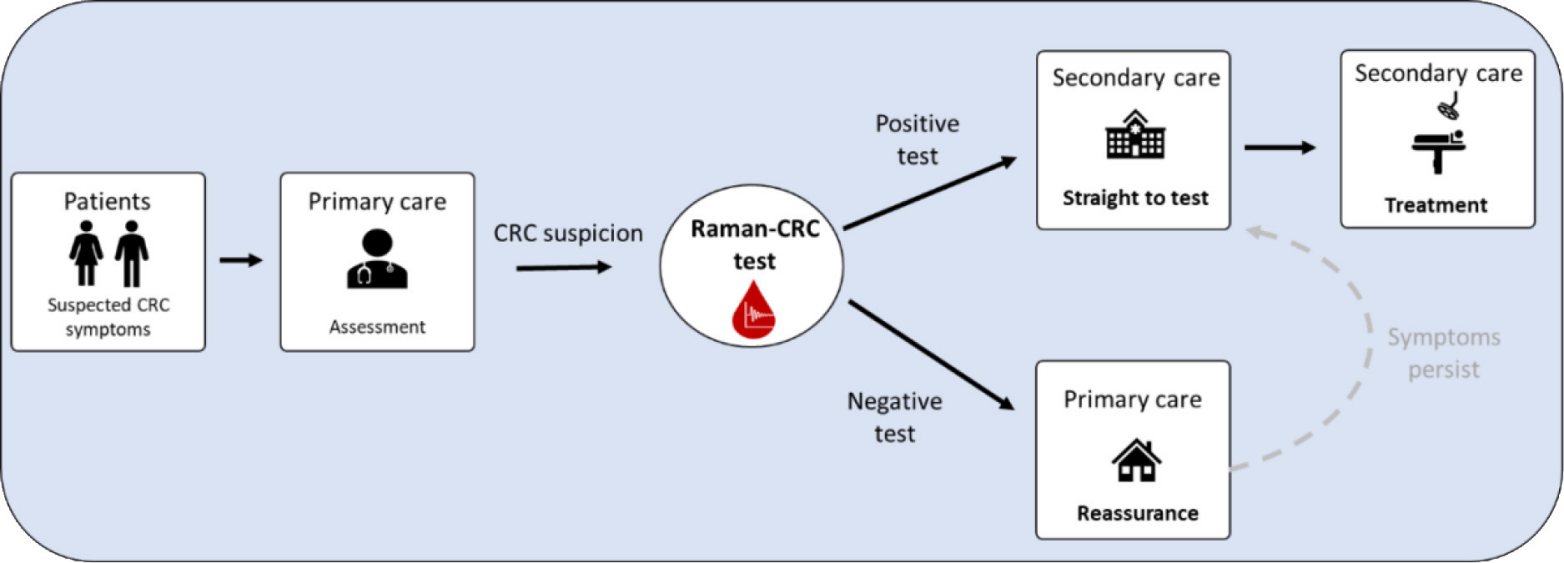
Proposed new clinical pathway incorporating Raman-CRC testing as a triage tool in primary care. Symptomatic patients with a negative Raman-CRC test are reassured in primary care, relieving pressure on secondary care diagnostic services. The pathway could lead to earlier diagnosis and a reduction in time to treatment when a positive test is combined with a straight to test pathway. CRC = colorectal cancer.

In conclusion, Raman-CRC has shown potential to become an acceptable decision support tool in primary care for symptomatic patients at risk of underlying CRC. The test is applicable to all relevant symptoms and could help upgrade patients with low-risk symptoms (including rectal bleeding) onto the USC pathway towards earlier detection. A positive test would circumvent the traditional referral route by dovetailing with a ‘Straight To Test’ pathway (Figure 3).^34^ The authors of the present study now plan to validate and expand the proof-of-concept findings through both the CRaFT study and further large-scale primary care trials.

## Data availability

The datasets generated and/or analysed during the current study are available within the article and supplementary information files are available from the authors upon reasonable request.

## Data

The dataset relied on in this article is available from the corresponding author on reasonable request.
